# Registration Difficulties—III

**Published:** 1908-11-21

**Authors:** 


### Nursing Administration.

### REGISTRATION DIFFICULTIES—III.

It has been shown that the theoretical objections to the registration of nurses rests on the grounds that examinations are not well adapted to determine the fitness of women to be nurses, and that a permanent State qualification conferred on nurses who satisfied certain conditions on the completion of their training, would add yet another embarrassment to the existing difficulties in distinguishing between good and bad nurses. Every matron can point to numerous excellent nurses who could never have succeeded in passing any examination of value considered as a standard. No matron could, with any confidence, declare that all the pupils whose certificates she signs at the conclusion of three or four years' training would still be efficient and trustworthy nurses after ten or fifteen years. Both these difficulties must be given their proper value in deciding whether it is well to institute a system of registration for nurses in general. The rights of those women whom registration would exclude from recognition must be considered, and the wrongs of those whom registration would recognise, but who should prove unworthy (not necessarily in the eyes of the law).

The ordinary definition of a training school for nurses is "a general hospital containing over one hundred beds." This is the line followed in the best known handbook to nurse-training schools, "How to Become a Nurse." * Many Poor-law infirmaries are no whit inferior to general hospitals as training schools, but their distinct organisation places them in a class by themselves. It may easily be argued that short of one hundred beds the hospital cannot afford complete opportunities to probationers for learning their business; that among a hundred beds 25 per cent. may often be empty, and that the remainder, divided up among male and female, surgical and medical, children, and isolation wards, supplies the very lowest amount of material which is necessary to make the pupil nurses efficient. All these arguments are unanswerable. It goes without saying that in general hospitals of at least one hundred beds the best training for nurses is to be had. Should the General Nursing Council lay it down as a preliminary to examination that a candidate should have been trained in a general hospital containing at least one hundred beds? There would be some justification for this course.

The number of pupils now under training as nurses in London general hospitals containing more than one hundred beds is 1,110, divided among sixteen institutions. In provincial general hospitals the numbers under training are 1,838, among fifty-nine institutions. In Scotch general hospitals the numbers are 703, among twelve institutions. In Irish general hospitals the numbers are 369, among ten institutions. The total number of nurses in training at the present moment in English, Scotch, and Irish general hospitals containing one hundred or more beds is, approximately, 4,020. These figures include pupil nurses in their first, second, and third years, whether classed as nurses or probationers, and, where the course extends to a fourth year, it includes the fourth year probationers also. The figures are, of course, approximate, for the number of probationers varies from year to year in all hospitals. They may be taken, however, as substantially correct, and should all these probationers complete their course with automatic regularity, it would give an average of about 1,200 trained nurses turned out per year by the large training schools, allowing for a three years' course in most hospitals and a four years' course in some. A wastage of about 10 per cent., however, has been found to operate pretty regularly among probationers, and that would reduce the actual number trained yearly in these institutions to about 1,100. This is the number of nurses who would be quite certain of being qualified to submit themselves for examination under any system of registration which could reasonably be devised. The nurses who have received this training are seldom at a loss in their future career. A large proportion of them remain in institution work, taking subsequent appointments in other hospitals with a view to matronships. Those who take up private work are speedily secured by reputable institutes or become attached to the private staffs of their own hospitals. Until they have passed their prime, unless impeded by defects of manner or character, they make a good income. It is in the interests of these nurses, and of that section of the public whom they serve, that registration is being demanded. But the public who can afford the services of nurses thus trained is only termed the "public" by courtesy. Needless to say it is not the 38,000,000 working people of these islands. It is doubtful if the luxury of the best nursing is within the reach of even the 3,750,000 classified by the political economists as "comfortable." The "public" here means the odd million who already have every facility for securing the best possible attendance in sickness as in health.

In effect the advocates of registration are demanding it in the interests of the smallest class in the community. They propose to draw a ring fence round the lucrative practice of nursing among people able to afford high fees, whereby this practice may be restricted to certain nurses. It may be a desirable thing to do. It is hardly a measure to excite much enthusiasm among those whose minds are set on a far more difficult problem. For the nurses trained under these conditions are quite insufficient to meet the demands of the real public for skilled nursing, in addition to all the requirements of institutions, and the proposed measure necessarily excludes from its scope both the nurses whose training is in most need of regulation, and the public which is in most need of nurses.

* "How to Become a Nurse: The Nursing Profession; How and Where to Train," edited by Sir Henry Burdett, K.C.B., K.C.V.O. (Scientific Press), price 2s. net.

